# Identification of Genes Associated with Nitrogen Stress Responses in Apple Leaves

**DOI:** 10.3390/plants10122649

**Published:** 2021-12-02

**Authors:** Youngsuk Lee, Van Giap Do, Seonae Kim, Hunjoong Kweon

**Affiliations:** Apple Research Institute, National Institute of Horticultural and Herbal Science, Rural Development Administration, 107, Soboangye-ro, Gunwi 39000, Korea; giapbio@korea.kr (V.G.D.); seonaekim@korea.kr (S.K.); kweonhj@korea.kr (H.K.)

**Keywords:** apple, nitrogen, transcriptome

## Abstract

Nitrogen (N) is an essential macronutrient that regulates diverse physiological processes for plant survival and development. In apple orchards, inappropriate N conditions can cause imbalanced growth and subsequent physiological disorders in trees. In order to investigate the molecular basis underlying the physiological signals for N stress responses, we examined the metabolic signals responsive to contrasting N stress conditions (deficient/excessive) in apple leaves using transcriptome approaches. The clustering of differentially expressed genes (DEGs) showed the expression dynamics of genes associated with each N stress group. Functional analyses of gene ontology and pathway enrichments revealed the potential candidates of metabolic signals responsible for N-deficient/excessive stress responses. The functional interactions of DEGs in each cluster were further explored by protein–protein interaction network analysis. Our results provided a comprehensive insight into molecular signals responsive to N stress conditions, and will be useful in future research to enhance the nutrition tolerance of tree crops.

## 1. Introduction

Nitrogen (N) is a fundamental macronutrient that plays an essential role in regulating a wide range of physiological metabolisms in plants for survival and development. Plants acquire N from the soil as inorganic forms via membrane transporters, such as ammonium transporters (*AMTs*) and nitrate transporters (*NRTs*) (or nitrate transporter/peptide transporters; *NPFs*) [1,2]. After uptake, N sources are utilized in plants, followed by N assimilation, which involves the reduction of nitrate to ammonium and conversion into amino acids [3,4]. The N availability and metabolic signaling of N assimilates are not only coordinated and balanced with internal carbon (C) metabolism during the developmental stages as a source–sink relationship [5,6,7], but are also primarily affected by the external conditions of N supply, including starvation/overabundance [8,9,10,11].

For many horticultural crops such as apple, the nutritional status of N affects many physiological processes. While insufficient N fertilization can cause decreased tree growth, reduction of sugar metabolism, or poor bud development [12,13,14], while the over-fertilization of N can cause a reduction in floral bud differentiation, alternate fruit-bearing, decrease in fruit quality, and elongation of vegetative growth [15,16,17,18,19].

Among the main horticultural crops, apple (*Malus domestica* Borkh.) occupies a large portion of the fruit industry in the world. In apple orchards, proper N fertilization is a critical component of farmers’ tree management, and during the growing season, the stress responses of apple trees induced by various N fertilization can result in unbalanced growth and physiological disorders. Global transcriptome data from previous studies [20,21] and recent studies have suggested examining the different responses to N tolerance in apple tissues [22,23]. However, little is known about the underlying molecular mechanisms of responsive metabolic signals associated with various N stress conditions in apple.

In order to investigate this further, we investigated molecular signals in response to different N stress (deficient/excessive) conditions using transcriptome analysis of apple leaves because the leaf tissue reflects the overall N nutritional status of apple trees during the growing season [24]. We identified the clusters of 2212 differentially expressed genes (DEGs), showing distinctive expression dynamics unique to each N stress. Functional enrichment and gene interaction network analyses indicated potential candidates for metabolic signals differentially associated with N stress conditions. Our results provided useful information about metabolic signals and the genes involved in N stress responses in apple.

## 2. Results and Discussion

### 2.1. Transcriptome Profiling and the Leaf N Content

**Figure 1:**
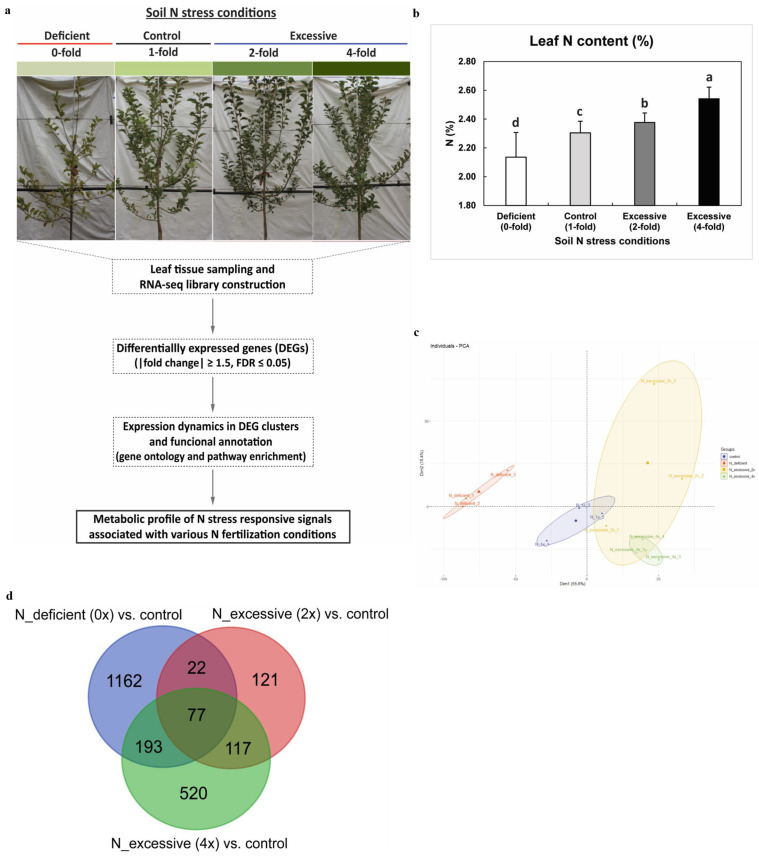
RNA-seq experiment design for the study of apple N stress response signals and DEG analysis. (**a**) A scheme for apple N stress response study. Leaf tissues of ‘Fuji’/M9 apple trees treated with different N fertilization gradients showing a different level of foliar N content were sampled for transcriptome analysis. (**b**) Leaf N content among four N fertilization groups. Lines on the bars show SE of the mean and different letters indicate significant difference as determined by DMRT (*p* < 0.05). (**c**) Principal component analysis of RNA-seq expression data. The top 5000 genes listed according to the variance rank were chosen as informative genes and used for PCA. (**d**) Venn diagram of DEGs with criteria of |fold change| ≥ 1.5 and FDR ≤ 0.05.

In this study, we investigated the transcriptome data of ‘Fuji’/M9 apple leaves from four different N fertilization groups consisting of two N stress conditions and the control: N-deficient (0×), control (1×), and N-excessive (2×, 4×) (Figure 1a). We observed significant differences (Duncan’s multiple range test, *p* < 0.05) in foliar N content among the four groups from the leaves collected from one-year-old shoots (Figure 1b). The N-excessive 4× group exhibited the highest foliar N content, followed by the 2×, control, and N-deficient 0× groups in a sequential manner, confirming that N fertilization treatments correlated with the divergent leaf N content in Fuji/M9 apple trees.

To study the metabolic signals associated with N stress responses in apple, we constructed RNA-seq profiles of leaf tissues in four N fertilization groups. A total of 647 million reads were obtained for 12 libraries across all groups and used for mapping against 45,116 protein-coding genes from the GDDH13v1.1 apple reference genome [25] (Tables S1 and S2). In the principal component analysis (PCA) on the data set of 12 libraries, PC1 explained 55.6% of the total variance, separating the difference among three N fertilization groups: N-deficient, control, and N-excessive, followed by the separation between 2× and 4× samples within the N-excessive group in PC2, showing 15.4% of the variance (Figure 1c). We did not observe a clear separation between the two groups of N-excessive (2×, 4×) in the first two PCs. However, this was expected, given that the leaf samples were collected from only the same Fuji genotype. We then identified a total of 2212 DEGs by comparing samples in N stress groups (0×, 2×, 4×) against the control (1×) group with a threshold of |fold change| ≥ 1.5 and FDR ≤ 0.05 (Figure 1d). Details of the DEGs in the N stress groups are presented in Table S3. The highest number of DEGs was detected in the N-deficient condition. The expression of identified DEGs was validated by qRT-PCR on a set of eight selected genes, where the relative expression pattern of qRT-PCR in the four N fertilization groups was compared with reads per kilobase per million (RPKM) data from RNA-seq (Figure S1; Tables S4 and S5). Together these results suggest that our transcriptome data reflect the samples collected from the contrasting N fertilization groups.

### 2.2. Analysis of Marker Genes Known to Be Associated with N Uptake, Assimilation, and Transport Pathways in Apple

**Figure 2:**
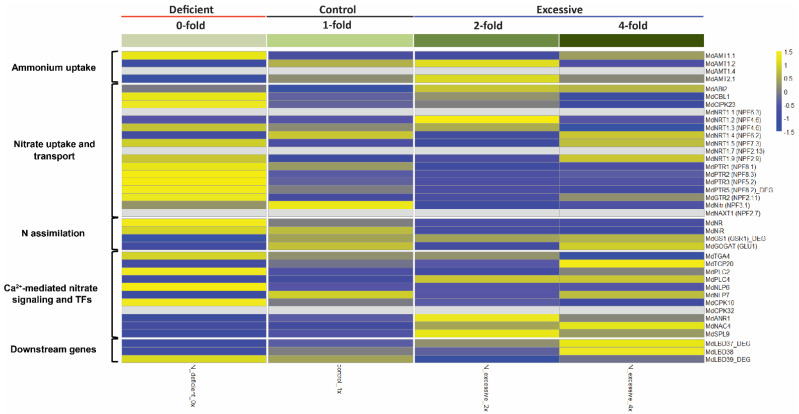
Expression profile of apple genes involved in a conserved N uptake, assimilation, and transport pathway.

In order to understand the dynamics of our transcriptome data associated with N stress responses in apple, we investigated the expression pattern of genes known to be involved in N uptake, transport, assimilation, and signaling pathways conserved in plant [1,2,26,27] In the ammonium uptake category, we observed that *MdAMT1.1* was notably upregulated in the N-deficient (0×) group. In contrast, its expression was low in control (1×) and N-excessive (2×) (Figure 2). Similarly, among the genes involved in nitrate uptake and transport, most genes of *MdNRTs* and *MdPTRs* were upregulated in the N-deficient group compared to others, including one *MdPTR5* gene that was also detected in our 2212 DEG set. On the other hand, the expression level of *MdNRT1.2* was rather maintained higher in the N-excessive (2×) group than in the other three groups, and *MdNitr* was downregulated in both the N-deficient and N-excessive groups, while the control (1×) showed upregulation of its expression. After the uptake of N from soil as ammonium or nitrate, plants utilize N through assimilation processes [3]. In our transcriptome data, there were clear differences in the expression patterns of N assimilation. Although *MdNR* and *MdNiR*, both of which are known to be involved in the primary nitrate assimilation pathway [28,29], were upregulated in the N-deficient group and downregulated in N-excessive groups, the expression of *MdGS1* and *MdGOGAT* genes, which are both involved in glutamate conversion from ammonia assimilation [30], showed a contrasting trend, contrary to the expression pattern of genes mediating nitrate assimilation with downregulation in N-deficient and upregulation in N-excessive groups.

After uptake into the cytoplasm, the process of nitrate signaling is mediated by Ca^2+^ [31]. As a primary nitrate response, Ca^2+^-CPK-NLP signaling is activated [32]. Our RNA-seq data showed a discrepancy among N stress groups. Although the expression pattern of *MdNLP6* and *MdNLP7* differed, *MdNLP6* rather showed a similar upregulation in the N-deficient group as that of the main CPK-NLP complex component *MdCPK10*. There were many TFs enriched instead of regulating Ca^2+^-mediated nitrate signaling that was found to be upregulated in the N-excessive group. In particular, these included the upregulation of *MdANR1*, a member of MADS box TFs acting as regulators of lateral root elongation [33], and *MdSPL9*, a TF that was proposed as a potential hub regulator in the plant N regulatory network [34] in N-excessive 2× followed by 4×, compared with the two remaining groups. Additionally, the expression of *MdNAC4*, another nitrate-responsive TF that also participates in nitrate response [35], was upregulated in the N-excessive group (highest in 4× followed by 2×). As downstream genes of the nitrate response, *LBD* (*LATERAL ORGAN BOUNDARY DOMAIN*) family *MdLBD37-39*, TFs that are known to negatively regulate anthocyanin biosynthesis [36] were enriched. The expression of *MdLBD37* and *MdLBD38* was upregulated in the N-excessive group, whereas the upregulation of *MdLBD39* was found in the N-deficient group. These results may imply that the response of N uptake is relatively triggered more under N starvation stress conditions, whereas the responses of N assimilation, signaling, and regulatory mechanisms are more likely to be activated under excessive N stress conditions.

### 2.3. Gene Expression Dynamics in N Fertilization Groups Associated with Different N Stress Responses

**Figure 3:**
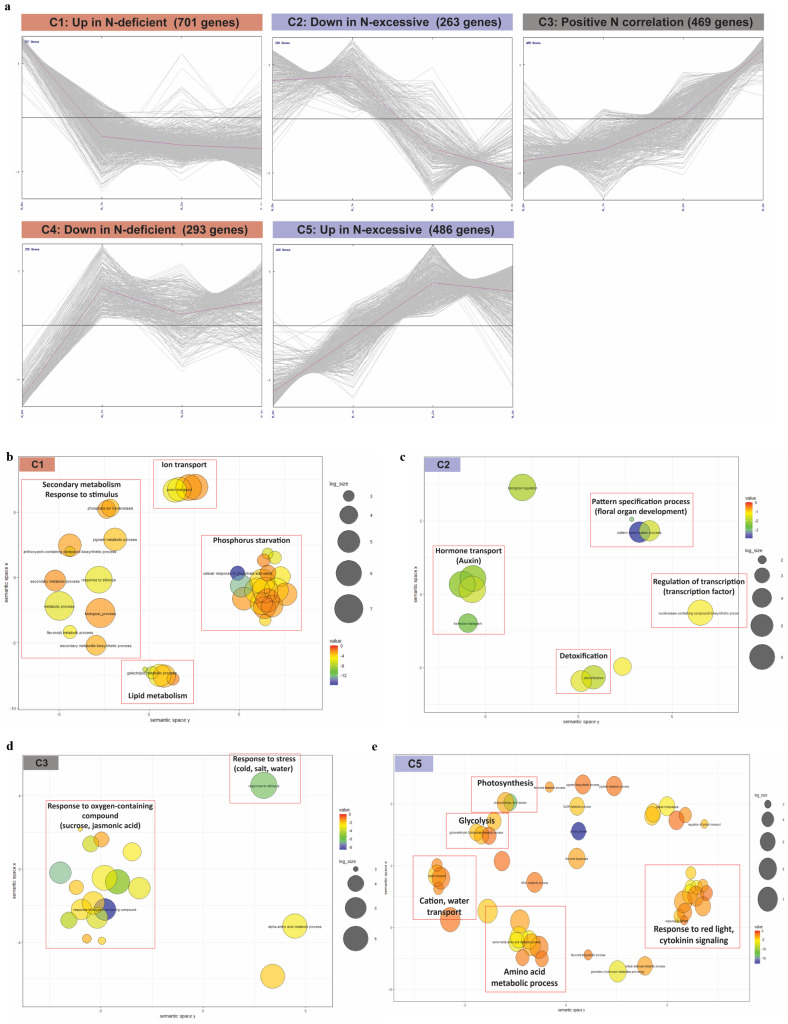
Investigation of DEGs functionally associated with nitrogen stress responses. (**a**) k-means clustering of rootstock 2212 DEGs with criteria of fold change above 1.5 and FDR below 0.05 (k = 5). (**b**–**e**) Gene ontology enrichment of DEGs in each cluster (biological process). All clusters except C4 (up in N-deficient) showed significant GO terms with a criteria of FDR 0.05. (**b**) C1: up in N-deficient; (**c**) C2: down in N-excessive; (**d**) C3: positive N correlation; (**e**) C5: up in N-excessive.

To uncover the expression patterns of genes responsive to N stress, we examined K-means clustering of 2212 DEG datasets, and five clusters were obtained (C1–C5, details are presented in Figure S2 and Table S6). Among the gene clusters, four clusters revealed the expression dynamics unique to each N stress condition (C1 and C4 specific to N-deficient; C2 and C5 specific to N-excessive), whereas cluster C3 was positively correlated with leaf N gradient (Figure 3a). In each cluster, we investigated the enriched GO terms (*FDR* < 0.05) (a detailed analysis is presented in Table S7). In cluster C1 (up in N-deficient), enrichment of the secondary metabolic process (GO: 0019748) and glycolipid metabolic process (GO: 0006664) were discovered (Figure 3b). In addition, the GO term response to stimulus (GO: 0050896) was also enriched, which included responses to osmotic stress and nutrients. Notably, phosphate starvation (GO: 0016036) was enriched in the form of clusters. This result may indicate that N-deficient nutritional conditions could trigger the defection of phosphorus (P) utilization in apple leaves stressed from N starvation, given that the uptake of N/P is known to be coordinated with each other mediated by integrative signals such as *NIGT1* and *SPX4* [37,38,39]. Compared to C1, cluster C2 (down in N-excessive) showed distinct GO enrichments different from those of C1. The main difference between C1 and C2 was the expression pattern of genes in control (1×). In C2, the main enrichments of GO terms were regulation of transcription (GO: 0006355) and detoxification (GO: 0098754) (Figure 3c). Furthermore, we found that the enrichment of auxin polar transport (GO: 0009926) was expected, considering that high nitrate conditions are known to repress auxin transport modulated by nitrate transporters [40]. Similarly, the enriched term floral whorl development (GO: 0048438) was discovered, suggesting that the process of floral organ development is downregulated under N-excessive stress conditions, which was partially supported by a previous study showing that *NRT1.1* controlled flowering time through the interaction with *FLC* signaling in plants [41].

In cluster C3 (positive N correlation), various GO terms were discovered, especially those related to stress responses, including abiotic stresses: water (GO: 0009415), cold (GO: 0009409), and chemicals: sucrose (GO: 0009744) and jasmonic acid (GO: 0009753) (Figure 3d). As previously reported, N fertilization could broadly affect stress responses, such as drought and defense in plants [42,43,44]. Since 469 DEGs in C3 represented the correlation to leaf N gradient in expression levels, we further investigated their correlation patterns in expression through a Pearson’s correlation analysis of gene expression with leaf N content across each N fertilization group (Table S8). Of the 469 DEGs, 347 genes showed a high correlation in their expression patterns with leaf N content (*r* ≥ 0.9).

On the other hand, no significant enriched terms (biological processes) were found in C4 (down in N-deficient). In cluster C5 (up in N-excessive), the main enriched GO terms contained photosynthesis (GO: 0015979) and response to carbohydrates (GO: 0009743) (Figure 3e), indicating that the process of generation and consumption of the energy cycle was strongly activated when N was over-fertilized. In terms of the relationship between photosynthesis and N response, the expression of the nitrate reductase (NR) gene is known to decrease when enough sucrose is produced by photosynthesis [45]. Our RNA-seq data indicated that a couple of NR genes, *MdNR* and *MdNiR,* were downregulated in the N-excessive group compared to the others (Figure 2), which may also support the enrichment result in C5.

### 2.4. Functional Annotation of DEGs in Five Clusters Associated with N Fertilization Stresses

**Figure 4:**
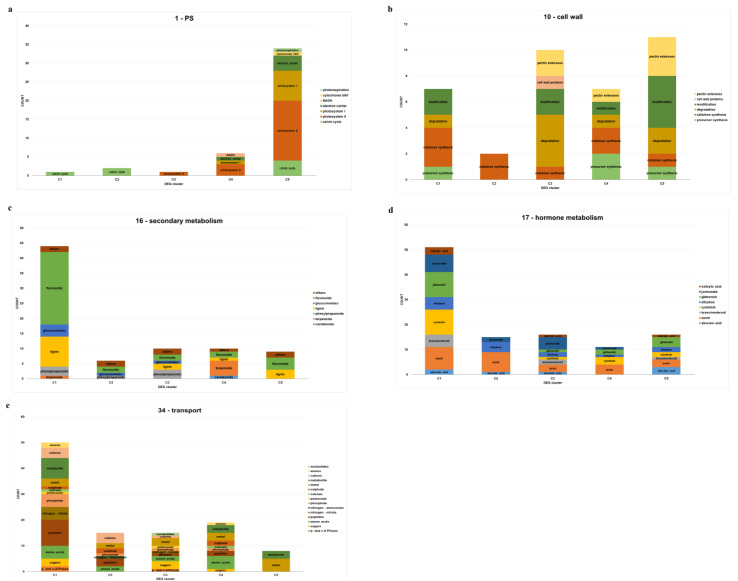
Functional annotation of DEGs: (**a**) photosynthesis; (**b**) cell wall; (**c**) secondary metabolism; (**d**) hormone metabolism; (**e**) transport.

We then investigated the five main categories of functionally annotated DEGs in each cluster, including photosynthesis, cell wall, secondary metabolism, hormone metabolism, and transport (Figure 4, Table S9).

In the category of photosynthesis, C5 (up in N-excessive) showed the largest enrichment among the five clusters, with more than 30 relevant genes associated with photosystems (I, II), electron carrier, Calvin cycle, and photorespiration. In comparison, the remaining four clusters showed enrichment of fewer than 10 genes (Figure 4a), indicating the functional importance of photosynthesis as an upregulated response in the N-excessive group. This result was concurrent with the discovery of the photosynthesis term (GO: 0015979) found in C5 (Figure 3e).

In the cell wall category, C3 (positive N correlation) and C5 revealed abundant enrichment relative to the others, showing cell wall modification and degradation-related genes occupying more than half of the entire gene count in each cluster (Figure 4b).

As for secondary metabolism, C1 (up in N-deficient) showed the largest enrichment of genes, most of which were involved in flavonoid and lignin biosynthesis (Figure 4c), which was concurrent with the GO enrichment of secondary metabolism (GO: 0019748) found in C1 (Figure 3b). Concerning flavonoids, we demonstrated that the expression of nitrate-responsive TFs *MdLBD37-38* was downregulated in N-deficient but upregulated in N-excessive groups (Figure 2), which repress anthocyanin biosynthesis [36]. Among the enriched genes related to lignin, *MdPAL* and *Md4CL* were found, the expression of which is also known to be induced under N-deficient conditions in tobacco as previously shown in [46] (Table S9).

In terms of hormone metabolism, we found a global enrichment of various hormones in most of the DEG clusters (Figure 4d). C1 showed the largest number of gene enrichments among the five clusters, while the remaining clusters showed less than 20 enriched genes. Gene ontology analysis revealed the enrichment of auxin in C2 and jasmonic acid in C3 (Figure 3c,d). The functional DEG annotation of C2 and C3 also showed a large proportion of auxin and jasmonic acid in each cluster, respectively.

In the transport group, various transporter-related genes were detected in the five clusters (Figure 4e). The DEG cluster exhibiting the largest enrichment of genes was C1, separated by 13 different target molecules in transport, but when compared to C4 (the other cluster representing N-deficient unique expression pattern contrary to C1), nitrate, potassium, and cation transporters were found to be unique to C1. On the other hand, comparing the list of enriched transporters between the pair of two contrasting N-excessive clusters (C2 and C5), ammonium, amino acids, cations, phosphate, and sulfate were C2-specific, while metabolites were unique to C5. These data suggested the possibility that global metabolic signals are differentially induced in response to nitrogen fertilization stress conditions in apple.

### 2.5. Identification of Candidate Metabolic Signals for N Stress Responses in Apple

We compared the KEGG pathway enrichments in each DEG cluster to further uncover the metabolic signals mediating N stress responses (Table S10). Among these, some metabolic pathways were commonly enriched in N-deficient/excessive clusters (e.g., nitrogen metabolism, carbon fixation, and α-linolenic acid metabolism), whereas others were specific to each DEG cluster (e.g., flavonoid biosynthesis, fatty acid elongation, and photosynthesis).

Among the N-deficient stress-associating clusters C1 (up in N-deficient) and C4 (down in N-deficient), flavonoid biosynthesis (ath00941) and carbon fixation (ath00710) were mainly enriched in C1, while C4 showed pathway enrichment of nitrogen metabolism (ath00910), α-linolenic acid metabolism (ath00592), and plant–pathogen interaction (ath04626). In particular, most of the enriched genes in the carbon fixation pathway were identified as the PPC phosphoenol pyruvate carboxylase (PEPC)-coding gene family (*MdPPC1*, *MdPPC3*, *MdPPC5*) (Table S11).

Among the enriched pathways found in N-excessive responsive DEGs C2 (down in N-excessive) and C5 (up in N-excessive), carbon fixation (ath00710) was enriched in both C2 and C5, and enrichment was also detected in C1. The functions of enriched genes within this category differed between C1 and C2. While *MdPPCs* were unique to C1, a couple of ribulose biphosphate carboxylase/oxygenase (Rubisco) small subunit (RBCS) family genes (*MdRBCS1A* and *MdRBCS3B*) were found to be specific to C2. Within the carbon fixation pathway, the expression of *MdRBCS1A* and *MdRBCS3B* genes is known to regulate the content of rubisco [47], the enzyme activity of which was reported to be reduced when its content was sufficient for photosynthesis under high N conditions in tree crops [48]. Considering these results, the enrichment of *RBCS* genes in N-excessive groups could be the result of the regulatory response triggered by the over-N condition to balance the excessive photosynthesis and N storage. On the other hand, apart from its anaplerotic function in carbon fixation, PEPC is also known to play a role in mediating the balance of carbon (C) and N metabolism by affecting both sugar accumulation and ammonium assimilation [49], suggesting that the enrichment of *PPCs* in the N-deficient group may reflect the physiological status of imbalanced C/N metabolism under N starvation stress condition. Furthermore, C5 showed enrichment of two photosynthesis-related pathways (ath00195, ath00196), ribosome (ath03010), N metabolism (ath00910), and hormone signal transduction (ath04075). Within the enrichment of photosynthesis-related pathways in C5, genes encoding photosystem I and II subunits (*MdPnsLs*, *MdPSAs*, *MdPSBs*) and antenna proteins (*MdLHCAs*, *MdLHCBs*) were upregulated in the N-excessive group, implying the maintenance of leaf photosynthesis under high N fertilization conditions.

Notably, although all clusters showed enrichment of hormone metabolism in the functional annotation of DEGs, most of which were biosynthesis-related (Figure 4e; Table S9), KEGG pathway analysis revealed that C5 exhibited a significant enrichment of hormone signal transduction (Tables S10 and S11). Auxin, cytokinin, and abscisic acid (ABA) hormones are involved in this signal transduction pathway, all of which are closely related to N signaling [50,51]. In the auxin signaling pathway, *MdTIR1*, a major auxin receptor that activates auxin responses [52] and a couple of Aux/IAA family genes (*MdIAA7* and *MdIAA16*) were discovered. The enrichment of cytokinin-signaling-related genes (*MdAARs*, *MdAHPs*) in N-excessive groups could be functionally linked to upregulation of *MdGS1* and *MdGOGAT* in our transcriptome data (Figure 2), provided that glutamate metabolism of N assimilation is known to be activated by cytokinin [53]. From the pathway enrichment of ABA signaling, we found that two genes were enriched in N-excessive groups, the main regulators of ABA signaling: a negative regulator *MdPP2CA* as the protein phosphatase 2C family, and a positive regulator *MdOST1* as the SnRK2 family. In addition to this, in our RNA-seq data, we demonstrated the downregulation of *MdCBL1* and *MdCIPK23* and the upregulation of *MdABI2* in N-excessive groups from the expression profile of genes involved in nitrate uptake and transport (Figure 2), suggesting that N uptake was suppressed under high N fertilization conditions. A recent study demonstrated that SnRK2-dependent ABA signaling repressed nitrate uptake [54], and the enrichment of *MdOST1* in the ABA signaling pathway may be concurrent with these expression data.

In cluster C3 (positive N correlation), we focused on the highly correlated 347 DEGs to analyze the KEGG pathway enrichment, since the expression pattern of these genes is expected to reflect the physiological changes according to the gradient of N fertilization conditions (Table S8). Interestingly, among the enriched metabolic pathways of highly correlated C3 DEGs, a couple of GAD family genes *MdGAD1* and *MdGAD4,* were found in the butanoate metabolism pathway that catalyzes the decarboxylation of glutamine to generate γ-aminobutyrate (GABA), implying that the conversion of glutamine to GABA might be functionally correlated with the external N gradient. GABA plays a role in balancing carbon and nitrogen metabolism [55,56]. Considering that GABA can also negatively control nitrate uptake under high N conditions [57], this butanoate pathway enriched in the highly correlated C3 DEG cluster might be linked to the downregulation of many nitrate-uptake-related genes, including the *MdPTR* family, in N-excessive groups compared to their upregulation in the N-deficient group (Figure 2).

**Figure 5:**
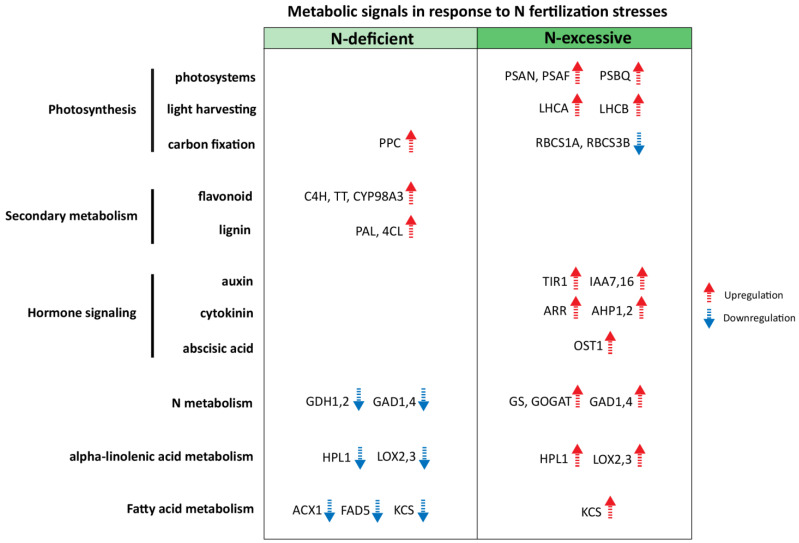
A proposed scheme for candidate metabolic signals associated with N-deficient/excessive stress responses in apple.

The metabolic candidate signals in response to N-deficient/excessive stress conditions are illustrated in Figure 5, integrated with the functional category of metabolic pathways. Our results provided a comprehensive overview of molecular characteristics for a detailed understanding of the underlying N nutritional signals in apple, implying the candidate genes to potentially target for further research to enhance N nutrition in apple.

### 2.6. PPI Network Analysis Provides the Additional Clue for Functionally Important Metabolic Signals Responsive to N Stresses

**Figure 6:**
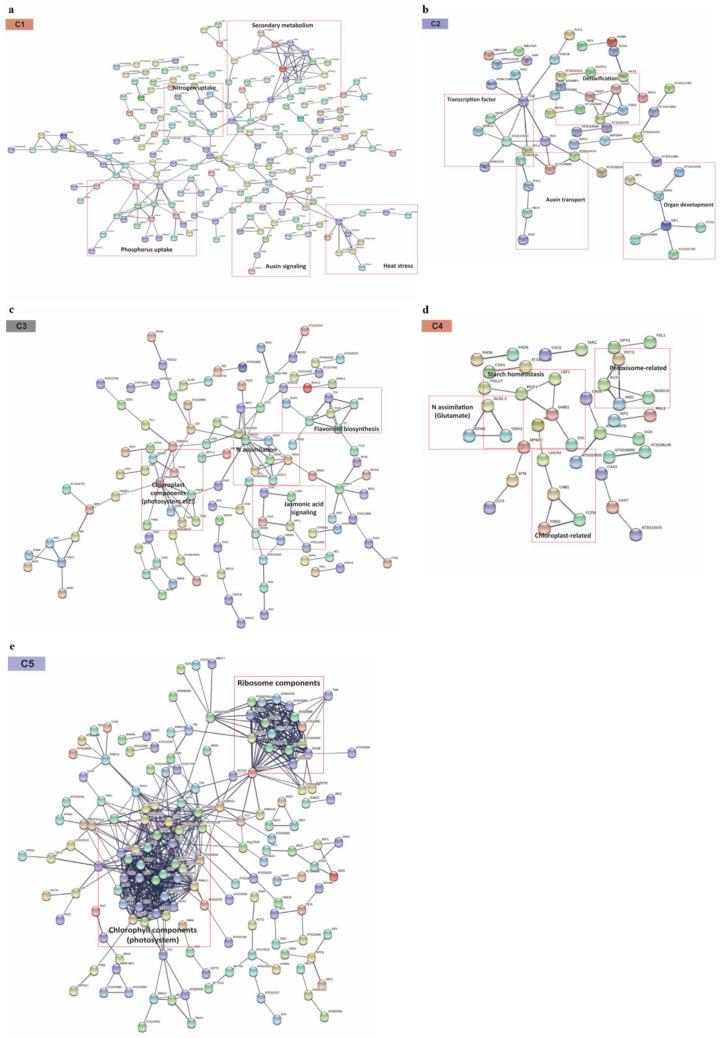
Network of protein–protein interaction (PPI) of five DEG clusters (C1–C5). (**a**–**e**) The PPI network in each DEG cluster was constructed with high confidence (0.7) using the STRING database: (**a**) C1, up in N-deficient; (**b**) C2, down in N-excessive; (**c**) C3, positive N correlation; (**d**) C4, down in N-deficient; (**e**) C5, up in N-excessive.

To examine another layer of evidence, we analyzed the PPI networks of the five DEG clusters (Figure 6). The interactions of gene pairs were compared in each DEG cluster to build the PPI networks at a high confidence level (combined score ≥ 0.7); among the five clusters, C5 showed the largest interaction pairs, while the rest of the networks in the four clusters contained much fewer interacting nodes (Table S12).

The PPI network of C1 (up in N-deficient) showed the interacting modules of genes involved in secondary metabolism, the uptake of N/P, auxin signaling, and heat stress (Figure 6a), which was also considerably concurrent with the GO enrichment results of the C1 cluster containing functional terms with secondary metabolic process (GO: 0019748), response to stimulus (GO: 0050896), and P starvation (GO: 0016036) (Figure 2; Table S7). Among the interacting modules in the PPI network of C2 (down in N-excessive), gene interactions mediating auxin transport, transcription factor, detoxification, and organ development were discovered (Figure 6b), in which metabolism was linked to GO term enrichment, representing detoxification (GO: 0098754), auxin transport (GO: 0060918), regulation of transcription (GO: 0006355), and floral whorl development (GO: 0048439) (Figure 2; Table S7).

The network of C3 DEGs (positive N correlation) contained the interacting modules involved in N assimilation, α-linolenic acid metabolism, and flavonoid biosynthesis (Figure 6c). Among these, α-linolenic acid metabolism was also discovered in the list of KEGG pathway enrichments from highly correlated C3 DEGs, including the relevant genes *MdHPL1* and *MdLOX2,3* involved in this pathway (Tables S10 and S11).

Although there were no significant GO terms found in C4 (down in N-deficient) (Figure 2), we found some interacting nodes in the C4 PPI network, which are expected to potentially function in organs of chloroplasts and peroxisomes, starch homeostasis, and N assimilation (Figure 6d). The PPI network of C5 (up in N-excessive) indicated the two main highly interacting clusters of gene modules that function in photosynthesis and ribosomal components (Figure 6e), suggesting the functional importance of the enrichment of metabolic pathways, including photosynthesis, the photosynthesis antenna protein, and the ribosome detected in C5 (Tables S10 and S11).

## 3. Materials and Methods

### 3.1. Plant Materials and Sample Collection

Fuji apple grafted onto M9 rootstocks were planted randomly onto 55 cm × 55 cm × 60 cm pots and were grown under natural field conditions in the experimental plot of the Apple Research Institute (36.3° N, 128.5° E), Gunwi, Korea. Apple trees were then divided into three groups according to levels of N fertilization: N-deficient (0×; without fertilization), control (1×; 7 g of urea), and N-excessive (2×, 14 g of urea; 4×, 28 g of urea). To study N-stress-responsive molecular signals in apple leaves, we collected foliar tissues between the third and tenth midshoot positions of new one-year-old shoots from each group after 6 months (Figure 1a) with four biological replicates.

### 3.2. Leaf N Content

Leaf samples were dried for 3 days and then ground. Contents from each ground leaf were extracted with a sulfuric acid solution with catalyst accelerators (K_2_SO_4_ + selenium (1000 Kjeltabs S/3.5, Foss, Hägersten, Sweden), heated at 420 °C for 1 h, and then cooled down for Kjeldahl digestion [58,59]. Leaf N content was measured using a Kjeldahl 8400 nitrogen analyzer (Foss, Hägersten, Sweden).

### 3.3. RNA Extraction and Sequencing

Total RNA was extracted using a modified CTAB method [59]. RNA quality was measured using an Agilent 2100 bioanalyzer (Palo Alto, CA, USA), and samples with a RIN of 7 or above were sent to CnK Genomics (Korea) for sequencing. There were three biological replicates in each N group used for sequencing. RNA-seq libraries were prepared using the MGIEasy RNA directional library preparation kit (MGI, Shenzhen, China), and a total of 12 libraries were constructed using the MGISEQ-2000 platform (MGI, Shenzhen, China) with 2 × 100 bp paired-end (PE) reads.

### 3.4. Read Processing, Mapping, and Expression Data Analysis

Raw reads were trimmed to remove low-quality and short sequences. Only PE reads with a quality score of <0.001 (equivalent to Phred Q30), ≤2 ambiguous nucleotides, and a length of ≥75 bp were retained for further analyses. Trimmed reads were aligned to the apple reference genome GDDH13v1.1 [25] in CLC Genomics Workbench 20 software [60] using the parameters of both the minimum length fraction and minimum similarity to 0.8. This genome version contained 51,000 genes, including 45,116 protein-coding genes and noncoding RNAs. Raw read counts were normalized to RPKM for each gene. Differentially expressed genes (DEGs) were determined by comparing three groups of N stress conditions (deficient: 0×; excessive: 2× and 4×) against control (1×) with a |fold change| ≥1.5, and a false discovery rate (FDR) value of ≤0.05. DEG clusters were analyzed using the K-means method in MeV software ver4.9.0 [61]. GO term enrichment was investigated using the agriGO enrichment tool [62] and clustered using the REVIGO visualization tool [63]. Pathway enrichment of DEGs was analyzed in each cluster using the DAVID bioinformatics resources database [64], based on the pairwise sequence comparison of GDDH13v1.1 transcript with NCBI Arabidopsis (TAIR10) homologs with a threshold of E-value < 1 × 10^−6^ provided from the GDR genome database [65].

### 3.5. Protein–Protein Interaction (PPI) Network Analysis

In order to build the PPI network of DEGs in each cluster, Arabidopsis homolog gene IDs were used to predict the PPI network using the STRING database [66]. The interaction score of each gene pair was obtained by combining the main sources (coexpression, lab experiments, text mining, and annotation in databases), and the PPI network was constructed with a high confidence level (combined interaction score ≥ 0.7).

### 3.6. Quantitative Reverse Transcription PCR (qRT-PCR)

First-strand complementary DNA was synthesized using 1.0 μg of total RNA, oligo dT primer, and Transcriptor Reverse Transcriptase (Roche, Penzberg, Germany). The qRT-PCR analysis was performed using LightCycler 480 SYBR Green I Master mix (Roche, Germany) on a Roche 480 LightCycler^®^ (Basel, Switzerland). Complementary DNA (1:20 dilution) was used as a template (5 μL) in a 20 μL reaction volume. For each sample type, there were 4–6 technical replicates. PCR cycles were as follows: initial denaturation at 95 °C for 5 min, followed by 45 cycles of 95 °C for 10 s, 65 °C for 15 s, and 72 °C for 12 s, and a final melt curve analysis to determine the amplification of a single product. Primers were designed using Primer-Blast (http://www.ncbi.nlm.nih.gov/tools/Primer-Blast, accessed on 10 May 2021) to span an intron, if possible, with a 100–150 bp product size. MDP0000336547 (SGF29 Tudor-like domain) was selected as the reference gene [67]. Primer efficiencies and relative expression levels of targets were calculated using the Roche 480 Light Cycler software E-Method [68].

### 3.7. Quantification and Statistical Analyses

The heatmap of genes showing hierarchical clustering was analyzed using the Pheatmap R package (https://cran.r-project.org/web/packages/pheatmap, accessed on 20 July 2021). Functional annotations were generated using the online Mercator v3.6 tool [69]. Other statistical analyses were performed using Microsoft Excel 2016 and the R program (http://www.R-project.org, accessed on 9 August 2021).

## 4. Conclusions

Recently, studies have targeted apple tissues to explore global N tolerance using RNA-seq analysis [22,23]; however, the detailed metabolic signals associated with N stress responses have not been investigated well. Our transcriptome data of apple leaves collected under contrasting N stress conditions provided valuable information about the expression dynamics of genes associated with each N-deficient/excessive stress response. The functional annotations of DEG clusters and pathway enrichment analyses demonstrated the potential list of regulatory genes that may contribute to the differential responses specific to contrasting N stress conditions, following further validation with the gene interaction network approach. In order to better understand the physiological responses to N stress, we should apply it to an orchard of fruit crops with improved fertilization or tolerance to external nutrient fluctuations. We suggest a collective profile of metabolic signals responsive to contrasting N stresses, including those related to photosynthesis (PSAF, LHCB, RBCS1A), hormone signaling (TIR1, ARR, OST) unique to N-excessive signals, and those related to carbon fixation (PPC) and secondary metabolism (C4H, PAL, 4CL) unique to N-deficient signals. Our results provided a novel insight into signals associated with N stress responses, and will be useful in future research to enhance N tolerance and optimize fertilization in apple.

## Data Availability

All sequencing data were deposited in the National Center for Biotechnology Information Sequence Read Archive database, bearing the BioProject ID PRJNA773514.

## Supplementary Materials

The following are available online at https://www.mdpi.com/article/10.3390/plants10122649/s1, Figure S1. The qPCR results for RNA-seq validation. RPKM data were compared with relative expression values in qPCR, and the correlation coefficient r was obtained using a set of 8 selected genes. Figure S2. Figure of merit (FOM) analysis of the 2212 DEG datasets. Table S1. Summary statistics of RNA-seq data. Table S2. Expression level (RPKM) of 45,116 apple genes in our transcriptome data. Table S3. DEGs from each N fertilization group and the expression level (RPKM) of 2212 pooled DEGs across all groups. Table S4. Primers used for qPCR validation. Table S5. The qRT-PCR results compared against RNA-seq data. Table S6. The five expression clusters of the 2212 DEG datasets (C1–C5). Table S7. GO enrichment analysis of five expression clusters (C1–C5). Table S8. Pearson’s correlation analysis of cluster 3 DEGs that positively correlated with leaf N content. Table S9. Functional annotation of DEGs in five expression clusters. Table S10. KEGG pathway enrichment of five expression clusters (C1–C5). Table S11. Annotation of genes found in the enriched metabolic pathways from five expression clusters (C1–C5). Table S12. Scoring of protein–protein interaction (PPI) of DEGs in five expression clusters (C1–C5).
