# Supplementary material for: Identification of Genes Associated with Nitrogen Stress Responses in Apple Leaves
Source: Plants (Basel). 2021 Dec 2;10(12):2649. doi: 10.3390/plants10122649 (PMC8706881; doi:10.3390/plants10122649)
Supplement: Supplementary file 1 [file plants-10-02649-s001.zip › Supporting_Information_Figures_nitrogen_stress_apple_revised_YL_vf.pdf]

Supporting Information Figure S1-S2

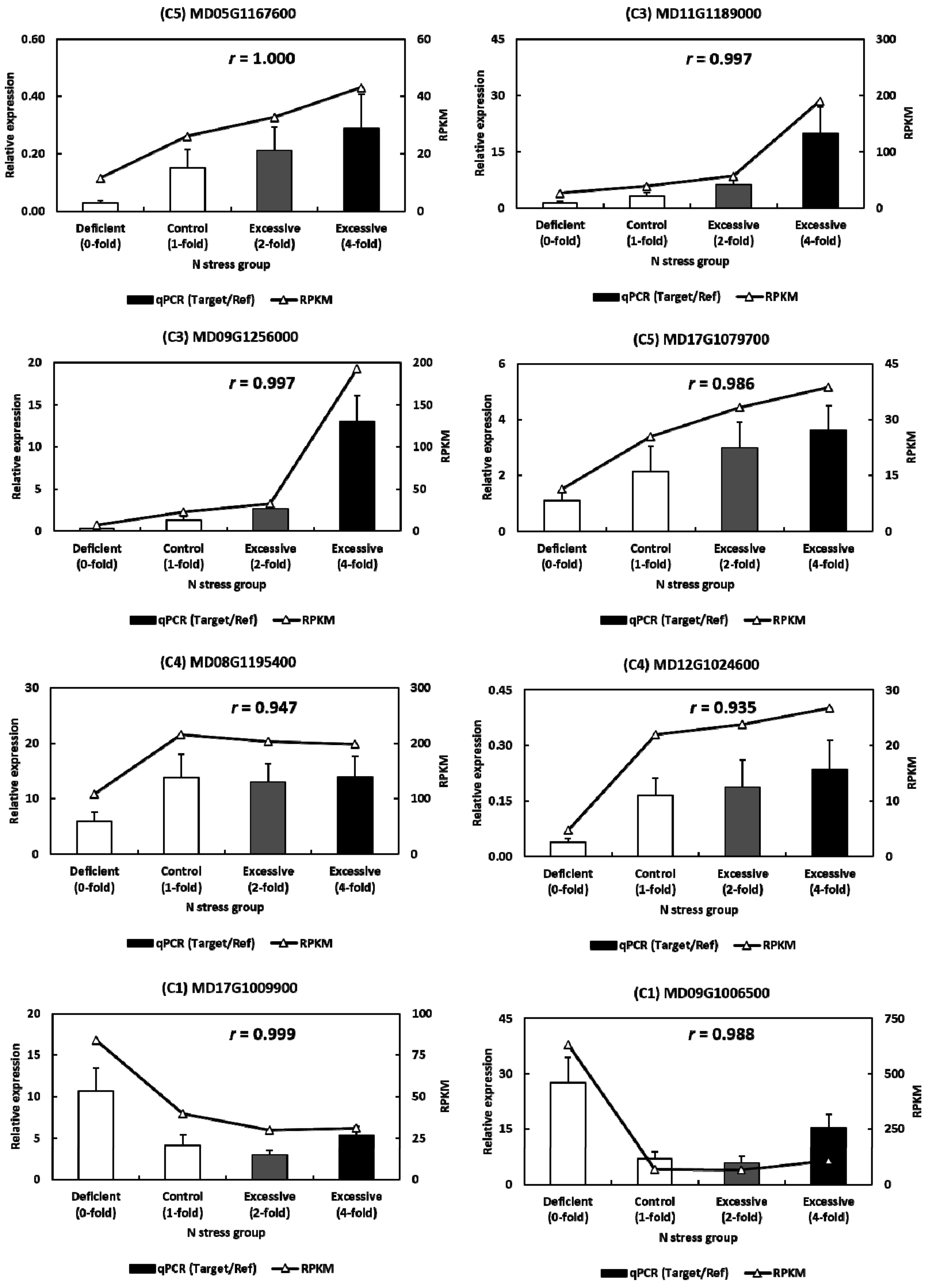

Figure S1. qPCR results for RNA-seq validation. RPKM data were compared with relative expression values in qPCR and the correlation coefficient *r* was obtained using a set of 8 selected genes.

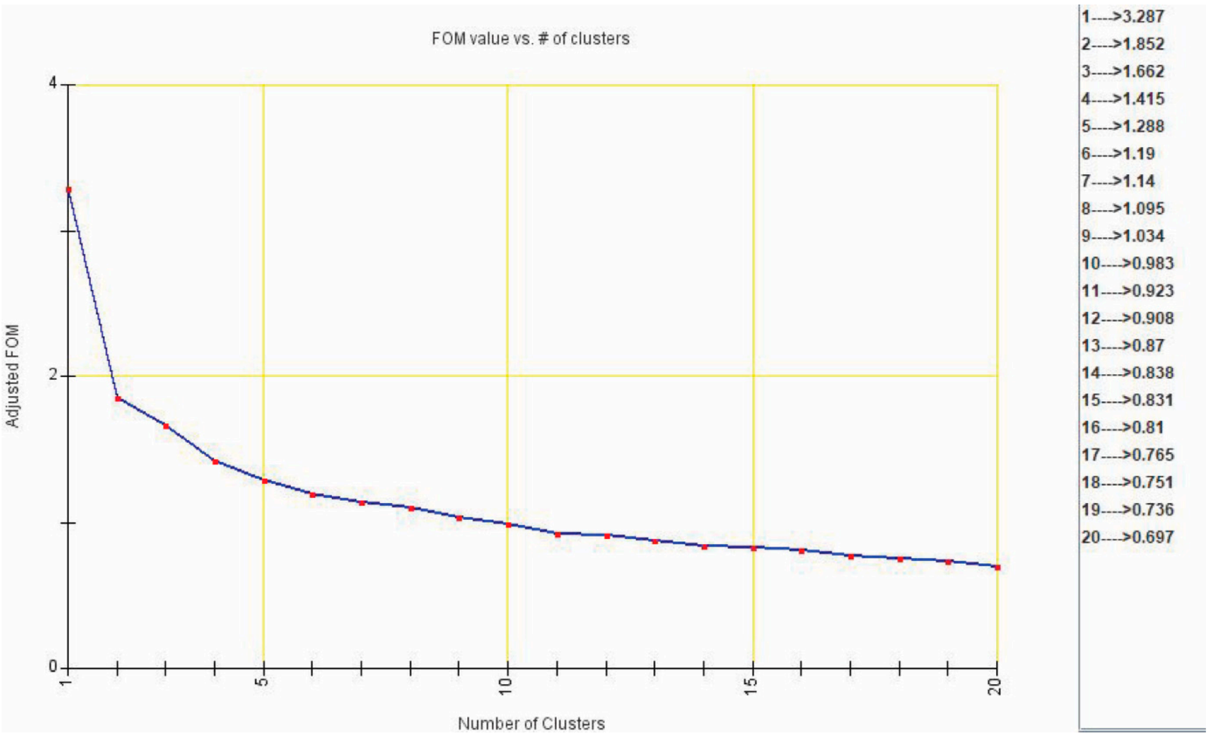

Figure S2. Figure of merit (FOM) analysis on 2,212 DEG dataset.
